# Ferroptosis-related protein biomarkers for diagnosis, differential diagnosis, and short-term mortality in patients with sepsis in the intensive care unit

**DOI:** 10.3389/fimmu.2025.1528986

**Published:** 2025-04-08

**Authors:** Zhangrui Zeng, Jie Deng, Gang Wang, Zixiang Luo, Weijia Xiao, Wenchao Xie, Jinbo Liu, Ke Li

**Affiliations:** ^1^ Department of Critical Care Medicine, The Second Affiliated Hospital of Xi’an Jiaotong University, Xi’an, China; ^2^ Core Research Laboratory, The Second Affiliated Hospital, Xi’an Jiaotong University, Xi’an, China; ^3^ Department of Laboratory Medicine, The Affiliated Hospital of Southwest Medical University, Luzhou, China; ^4^ Sichuan Province Engineering Technology Research Center of Clinical Diseases Molecular Diagnosis, Luzhou, China; ^5^ Clinical Diseases Molecular Diagnosis Key Laboratory of LuZhou, Luzhou, China

**Keywords:** sepsis, ferroptosis-related proteins, ACSL4, GPX4, PTGS2, diagnosis, prognosis

## Abstract

**Background:**

Sepsis is a disease with high mortality caused by a dysregulated response to infection. Ferroptosis is a newly discovered type of cell death. Ferroptosis-related genes are involved in the occurrence and development of sepsis. However, research on the diagnostic value of ferroptosis-related protein biomarkers in sepsis serum is limited. This study aims to explore the clinical value of Ferroptosis-related proteins in diagnosing sepsis and predicting mortality risk.

**Methods:**

A single-center, prospective, observational study was conducted from January to December 2023, involving 170 sepsis patients, 49 non-septic ICU patients, and 50 healthy individuals. Upon ICU admission, biochemical parameters, GCS, SOFA, and APACHE II scores were recorded, and surplus serum was stored at -80°C for biomarker analysis via ELISA. Diagnostic efficacy was evaluated using ROC curve analysis.

**Results:**

Baseline serum levels of ACSL4, GPX4, PTGS2, CL-11, IL-6, IL-8, PCT, and hs-CRP significantly differed among sepsis, non-septic, and healthy individuals (all p*-*value *<* 0.01). ACSL4, GPX4, PTGS2, IL-6, IL-8, PCT, and hs-CRP demonstrated high diagnostic and differential diagnostic performance (AUC: 0.6688 to 0.9945). IL-10 and TNF-α showed good diagnostic performance (AUC = 0.8955 and 0.7657, respectively). ACSL4 (AUC = 0.7127) was associated with predicting sepsis mortality. Serum levels of ACSL4, CL-11, and IL-6 above the cut-off value were associated with shorter survival times. ACSL4 levels were positively correlated with SOFA (Rho = 0.354, p*-*value < 0.0001), APACHE II (Rho = 0.317, p*-*value < 0.0001), and septic shock (Rho = 0.274, p*-*value = 0.003) scores but negatively correlated with the GCS score (Rho = -0.218, p*-*value = 0.018). GPX4 levels were positively correlated with SOFA (Rho = 0.204, p*-*value = 0.027) and APACHE II (Rho = 0.233, p*-*value = 0.011) scores.

**Conclusion:**

ACSL4 and GPX4 have strong diagnostic and differential diagnostic value in sepsis, including the ability to predict 28-day mortality in sepsis patients, and may become new potential serum markers for the diagnostic and differential diagnostic of sepsis.

## Introduction

Sepsis is a life-threatening condition characterized by a dysregulated response to infection, ultimately leading to organ dysfunction (1). It is also a highly heterogeneous clinical syndrome, with significant variability observed at the individual patient level, This heterogeneity poses substantial challenges in clinical management (2, 3). Current challenges in the clinical management of sepsis include poor treatment outcomes and high mortality, mainly due to unclear pathogenesis and the lack of effective diagnostic, therapeutic, and prognostic markers, especially those that can identify sepsis early with good sensitivity (4). An increasing number of studies have highlighted that pathogens can manipulate ferroptosis - a form of regulated cell death - to facilitate their replication, spread, pathogenicity, and evasion of the host’s immune response, thereby enhancing their survival (5). Recent research has also revealed a strong association between ferroptosis and the occurrence and progression of sepsis (6). Ferroptosis appears to play a crucial role in modulating inflammation and sepsis, and ferroptosis inhibitors have been shown to mitigate sepsis-induced multiple organ dysfunction and inflammation (7, 8). Given these findings, could ferroptosis-related markers emerge as novel diagnostic or prognostic indicators for sepsis?

Ferroptosis is a distinct form of iron-dependent programmed cell death. Unlike apoptosis, autophagy, and necrosis, ferroptosis is characterized by the excessive accumulation of iron-dependent lipid peroxides, ultimately leading to cell death (9). While previous studies have predominantly focused on ferroptosis in malignant tumors, its role in pathogen infection has been significantly underestimated (10–12). However, emerging evidence now highlights a close relationship between pathogen infection and ferroptosis (13–17). In sepsis models, glutathione peroxidase 4 (GPX4) exerts a pivotal role in inhibiting ferroptosis, thereby mitigating sepsis-induced multi-organ damage (18, 19). By neutralizing lipid reactive oxygen species (ROS) and preserving cellular integrity, GPX4 acts as a critical safeguard against oxidative stress and iron-dependent lipid peroxidation that characterize ferroptosis. This protective mechanism is particularly vital in the context of sepsis, where systemic inflammation and disrupted iron homeostasis create a milieu conducive to ferroptotic cell death. Targeting the GPX4 pathway may thus offer therapeutic potential for attenuating the severity of sepsis and its associated organ dysfunction. Therefore, GPX4 plays a crucial role in inhibiting pathogen infection and holds potential value as an infection marker (20).

Lipid oxidation is another significant factor in ferroptosis (21), Acyl-CoA synthetase long-chain family member 4 (ACSL4) enhances the production of polyunsaturated fatty acid-containing phospholipids (PUFA-PL), which in turn promotes the synthesis of lipid ROS. This process is a hallmark of ferroptosis (5, 22). In sepsis, vascular dysfunction is characterized by increased vascular permeability and pericyte loss, with pericytes being essential for maintaining vascular integrity. Recent studies have demonstrated that downregulation of ACSL4 significantly reduces lipopolysaccharide (LPS)-induced lipid peroxidation, restores pericyte viability, and improves endothelial permeability. *In vivo* experiments using pericyte-specific ACSL4 knockout mice showed marked reductions in pericyte loss and enhanced vascular barrier function following sepsis induction (23, 24). Collectively, these findings establish ACSL4 as a central mediator of ferroptosis in sepsis-associated pericytes, suggesting its potential as a diagnostic biomarker for infectious pathologies. Prostaglandin-endoperoxide synthase 2 (PTGS2/COX2) is well-known for its role in metabolizing arachidonic acid (AA) into prostaglandins. It is widely used as a ferroptosis biomarker in both *in vitro* and *in vivo* studies (20, 25, 26). ACSL4 and PTGS2 proteins have been employed to monitor ferroptosis responses through Western blot analysis, immunohistochemistry, or immunofluorescence analysis (21).

In recent years, the role of ferroptosis in sepsis has garnered increasing attention; however, its precise mechanisms remain to be fully elucidated. Identifying ferroptosis-related proteins associated with sepsis may pave the way for novel approaches in early diagnosis and treatment. To this end, this study aims to select a panel of potential inflammatory factors, including ferroptosis proteins (ACSL4, GPX4, and PTGS2), chemokines, cytokines, and collectin-11(CL-11) and investigate the clinical value of these proteins in the diagnosis and prognosis of sepsis, as well as predicting mortality risk in a newly established cohort of sepsis patients. Ultimately, this study seeks to provide guidance for future clinical research on sepsis management and identify potential diagnostic targets.

## Materials and methods

### Study population and design

**Figure 1:**
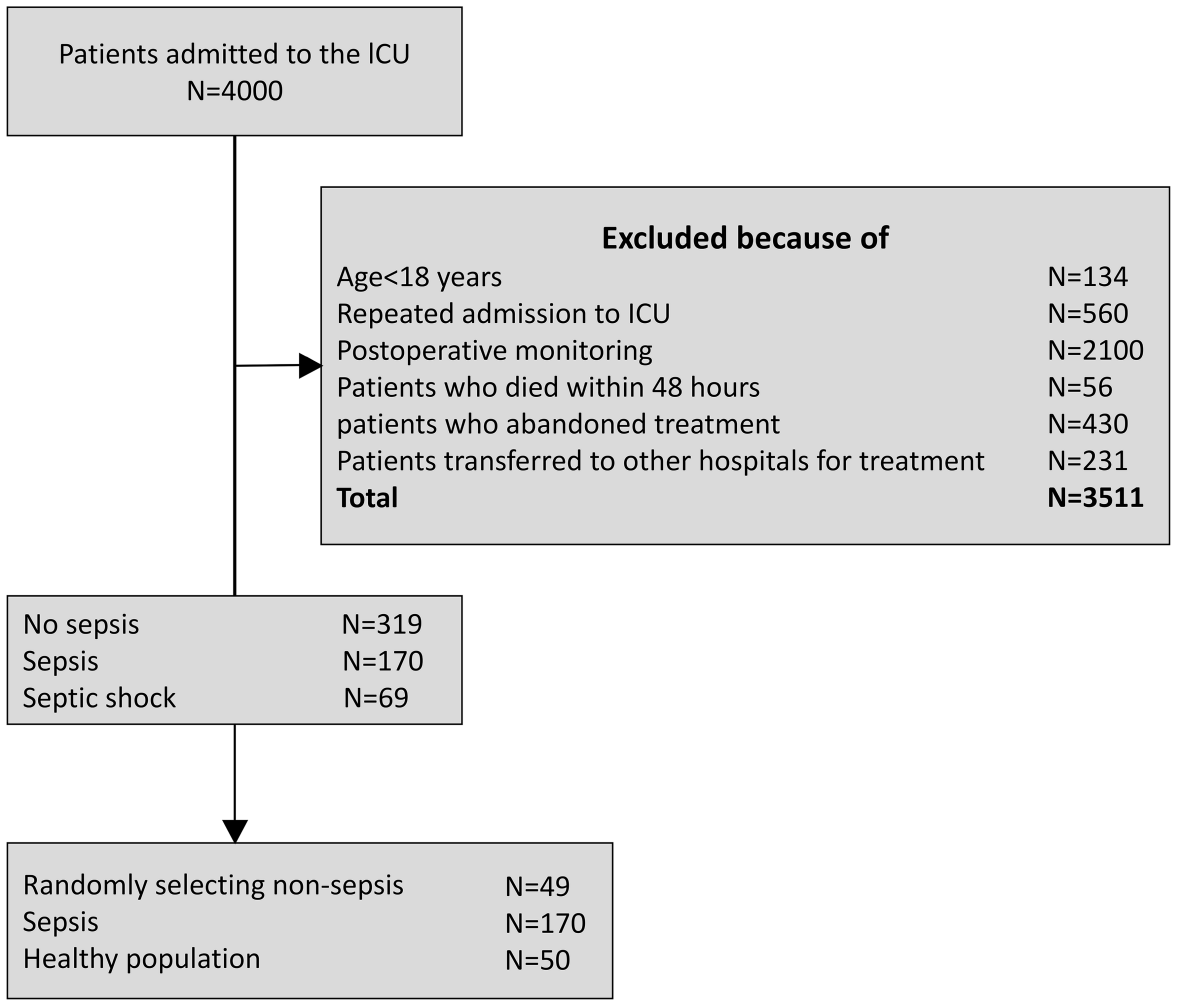
Flowchart of study design and patient selection.

This is a single-center, prospective, observational study. **Figure 1** presents a flowchart of the study design and exclusion criteria. The study population included all adult patients (>18 years old) admitted to the intensive care unit (ICU) between January 1, 2023, and December 31, 2023 (a 12-month period) who were eligible for inclusion after screening. Patients who were readmitted to the ICU were only registered based on their first ICU admission. The following exclusion criteria were applied: admission solely for postoperative monitoring; death within 48 hours of ICU admission; and patients who either abandoned treatment or were transferred to other hospitals for further care. Data were initially recorded in the case record form by the attending ICU physician and the project researchers. The final discharge status of all admitted patients was obtained from the hospital medical record system. Healthy controls were recruited from individuals who had normal findings in physical examinations conducted at the Health Management Centre of the Affiliated Hospital of Southwest Medical University. In this study, patients were assigned to the sepsis group according to the definitions provided in the Third International Consensus Definitions for Sepsis and Septic Shock (Sepsis-3) guidelines (1).

### Ethical review

This study protocol was approved by the Clinical Trial Ethics Committee of the Affiliated Hospital of Southwest Medical University (Project Number: KY2022267). The clinical trial is registered under the number ChiCTR2500096441. The Clinical Trial Ethics Committee of the Affiliated Hospital of Southwest Medical University is responsible for overseeing the implementation of this project.

### Data collection

Upon admission to the ICU, blood analysis was conducted as part of the routine clinical diagnosis and treatment plan. This included measurements of white blood cell count (WBC), neutrophil percentage (NEU%), blood lactate (LA), glomerular filtration rate (GFR), and infection biomarkers such as high-sensitivity C-reactive protein (Hs-CRP) and procalcitonin (PCT). These tests were performed in the hospital’s medical laboratory. The remaining serum or plasma after the tests were collected and stored in an ultra-low temperature freezer at -80°C.To confirm the diagnosis of sepsis, bacterial cultures were obtained from blood, nasopharyngeal swabs, urine, and tracheal aspirates or sputum upon admission. These samples were processed according to the standardized protocols of the clinical microbiology department. The final discharge status of all admitted patients were obtained from the hospital medical record system. The collected information included the patient’s name, age, gender, personal history, medical history, and disease status. Additionally, data on vital signs at the time of admission (such as blood pressure and temperature) were recorded, along with the Sequential Organ Failure Assessment (SOFA) score, the Acute Physiology and Chronic Health Evaluation (APACHE II) score, and the Glasgow Coma Scale (GCS) score. In the event of death, the date of death was also registered.

### Enzyme-linked immunosorbent assays

The levels of tumor necrosis factor-alpha(TNF-α), interleukin-1beta(IL-1β), IL-6, IL-8, IL-10, ACSL4, GPX4, C-X-C motif chemokine ligand 2(CXCL2), monocyte chemotactic protein-1(MCP-1), CL-11, and PTGS2 (COX-2) in the serum were measured using enzyme immunoassay kits, following the procedures described by the manufacturers’ instructions. The kits for TNF-α (KE00154), IL-1β (KE00021), IL-6 (KE00139), IL-8 (KE00006), IL-10 (KE00170), and MCP-1 (KE00091) were sourced from Proteintech, China. The ACSL4 (EH6088) and GPX4 (EH8916) kits were obtained from Wuhan Fine Biotech Co., Ltd., China. The CXCL2 (MM-0289H2) kit was from MEIMIAN, China. The CL-11 (E-EL-H6051) and PTGS2 (COX-2) (E-EL-H1846c) kits were from Elabscience, China. The absorbance intensities were measured at 450 nm using the EnSpire Multimode Plate Reader (PerkinElmer Inc., USA) and data was exported using Microsoft Excel Office 2019 software and analyzed using GraphPad Prism version 10 software package.

### Outcome measures

Our primary analysis focused on the relationship between baseline infection marker levels and short-term mortality. We compared the serum marker levels (expressed as medians) between patients with sepsis who survived and those who died within 28 days. Additionally, we assessed the illness severity by analyzing the serum levels of infection markers in patients with septic shock versus those with sepsis.

### Statistical analyses

Categorical variables were compared using the chi-square test or Fisher’s exact test, as appropriate. Continuous variables were analyzed using Student’s t-test for normally distributed data or the Mann–Whitney U test for non-normally distributed data. Correlations were assessed using Spearman’s rank correlation test for non-parametric data and point-biserial correlation analysis for mixed data types. Survival rates were evaluated using the Kaplan-Meier estimator, with time-to-event analysis measuring the interval from ICU admission to death from any cause within 28 days. Both univariate and multivariable Cox proportional hazards regression analyses were conducted to evaluate 28-day mortality, with results reported as hazard ratios (HR) and 95% confidence intervals (CI). All variables included in the Cox regression model satisfied the proportional hazards assumption, as confirmed by Schoenfeld residual testing. Receiver operating characteristic (ROC) curve analysis was performed to assess the predictive performance for 28-day mortality and disease severity (septic shock vs. non-septic shock). Comparisons among groups were performed using one-way ANOVA or the Kruskal-Wallis test. Statistical significance was defined as a two-tailed p-value < 0.05. All statistical analyses were used with IBM SPSS Statistics version 27 for Windows (SPSS Inc., Chicago, IL, USA). The figures for all data were created using GraphPad Prism version 10(GraphPad Software, La Jolla, CA, USA).

## Results

### Analysis of clinical basic characteristics

Between January 1, 2023, and December 31, 2023, a total of 4,000 patients were admitted to the intensive care unit (ICU). After applying the study’s inclusion criteria, 3,511 patients were excluded, leaving 489 eligible participants (**Figure 1**). Among these, 319 patients (65.2%) did not meet the diagnostic criteria for sepsis, while 170 (34.8%) were diagnosed with sepsis, including 69 (14.1%) who met the criteria for septic shock. from the 319 non-septic patients, 49 were randomly selected as non-septic controls. Additionally, 50 healthy adults were enrolled to form the healthy control group for this cohort study. Baseline characteristics of the entire cohort, as well as subgroup analyses of survivors and non-survivors based on 28-day mortality rates, were evaluated. Detailed results of this analysis are provided in **Supplementary Table 1**.

### The serum levels of ferroptosis-related proteins among the three groups exhibited statistically significant difference

**Figure 2:**
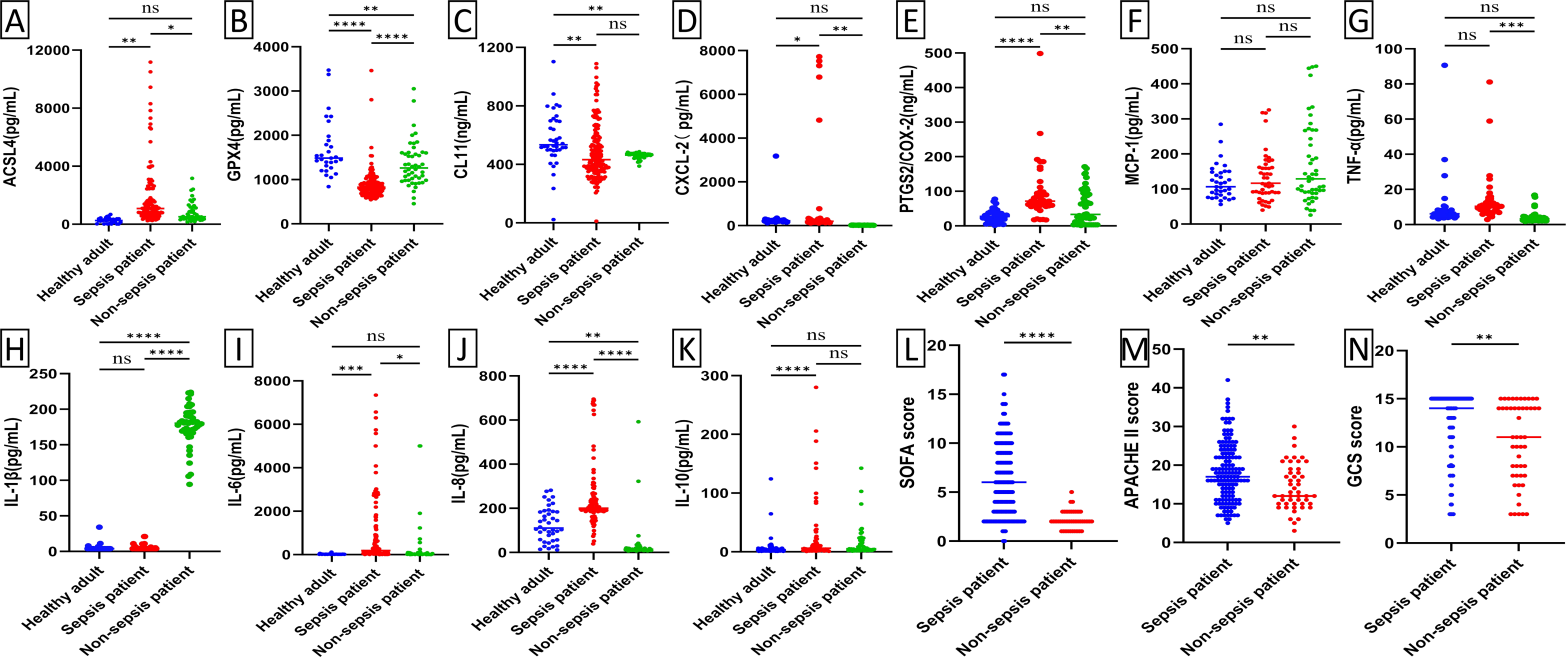
Scatter plot of serum biomarker levels and ICU clinical scores in healthy subjects (n=50), ICU patients with sepsis (n=170), and ICU patients without sepsis (n=59). Differences between median levels of **(A)** ACSL4, **(B)** GPX4, **(C)** CL11, **(D)** CXCL2, **(E)** PTGS2, **(F)** MCP-1 **(G)** TNF-α, **(H)** IL1β, **(I)** IL-6, (J) IL-8, **(K)** IL-10, **(L)** SOFA score, **(M)** APACHE II score and **(N)** GCS score in the three groups were analyzed by the Student's t-test or the Mann -Whitney U test. Ns, No statistical significance.

**Figure 3:**
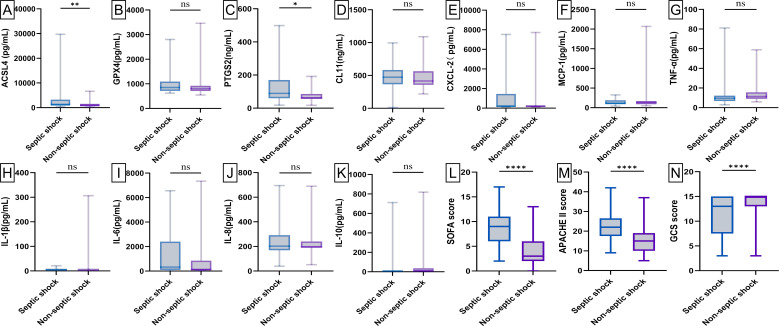
Box plot of serum biomarker levels and ICU clinical scores in patients with septic shock (n = 69) and septic non-shock (n = 101). Differences between median levels of **(A)** ACSL4, **(B)** GPX4, **(C)** PTGS2, **(D)** CL11, **(E)** CXCL2, **(F)** MCP-1, **(G)** TNF-α, **(H)** IL1β, **(I)** IL-6, **(J)** IL-8, **(K)** IL-10, **(L)** SOFA score, **(M)** APACHE II score and **(N)** GCS score in the two groups were analyzed by the Student's t-test or the Mann -Whitney U test. A record of missing values is available in **Supplementary Figure 3**. ns, No statistical significance. *p-value < 0.05, **p-value < 0.001,****p-value < 0.00001.

**Figure 4:**
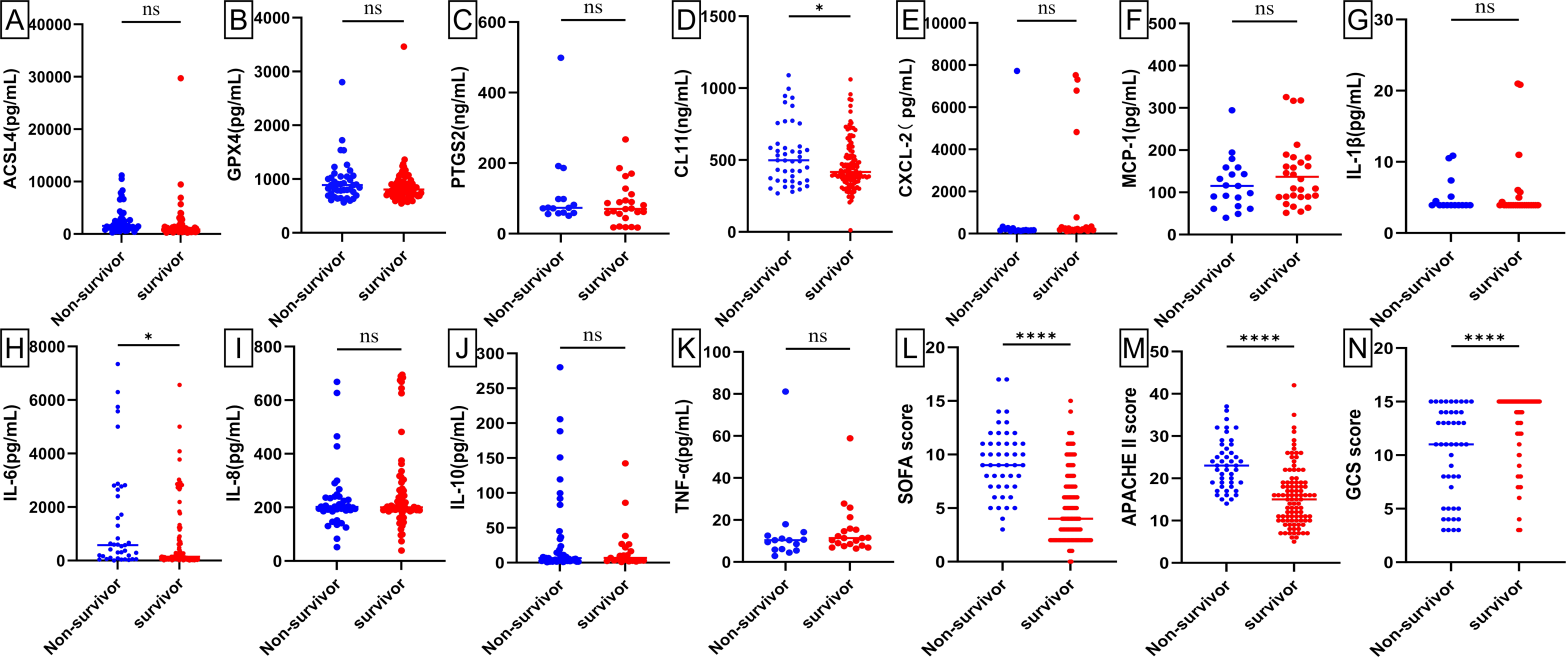
Scatter plots of serum biomarker levels and ICU clinical scores in sepsis survivors (n = 124) and sepsis non-survivors (n = 46). Differences between median levels of **(A)** ACSL4, **(B)** GPX4, **(C)** PTGS2, **(D)** CL11, **(E)** CXCL2, **(F)** MCP-1 **(G)** IL1β, **(H)** IL-6, **(I)** IL-8, **(J)** IL-10, **(K)** TNF-α, **(L)** SOFA score, **(M)** APACHE II score and **(N)** GCS score in the two groups were analyzed by the Student's t-test or the Mann -Whitney U test. A record of missing values is available in **Supplementary Figure 4**. ns: No statistical significance.

Baseline levels of biochemical markers were analyzed using enzyme-linked immunosorbent assay (ELISA) in septic patients, non-septic ICU patients, and healthy individuals (**Figure 2**; **Supplementary Figure 1**). Septic patients had higher levels of ACSL4, PTGS2, IL-6, IL-8, PCT, WBC, and hs-CRP, but lower levels of GPX4, CXCL-2, CL-11, GFR, and NEU% compared to healthy individuals (all p*-*value <0.00001-0.05). Compared to non-septic patients, septic patients exhibited higher levels of ACSL4, PTGS2, IL-6, IL-8, PCT, CXCL-2, and hs-CRP (all p*-*value <0.00001-0.01), but lower levels of GPX4, IL-1β, GFR, and NEU% (all p*-*value <0.00001-0.001). Among patients with septic shock versus those without (**Figure 3**; **Supplementary Figure 2**), baseline serum levels of ACSL4, PTGS2, and LA were significantly higher in those with septic shock. No significant differences were observed in other biomarkers (p*-*value > 0.05). In comparisons between septic survivors and non-survivors (**Figure 4**; **Supplementary Figure 3**), CL-11 and IL-6 levels were significantly higher in non-survivors, while neutrophil percentage (NEU%) was significantly lower. No significant differences were found in other biomarkers (p*-*value > 0.05).

### Ferroptosis-related proteins exhibit substantial diagnostic and differential diagnostic value in sepsis

**Figure 5:**
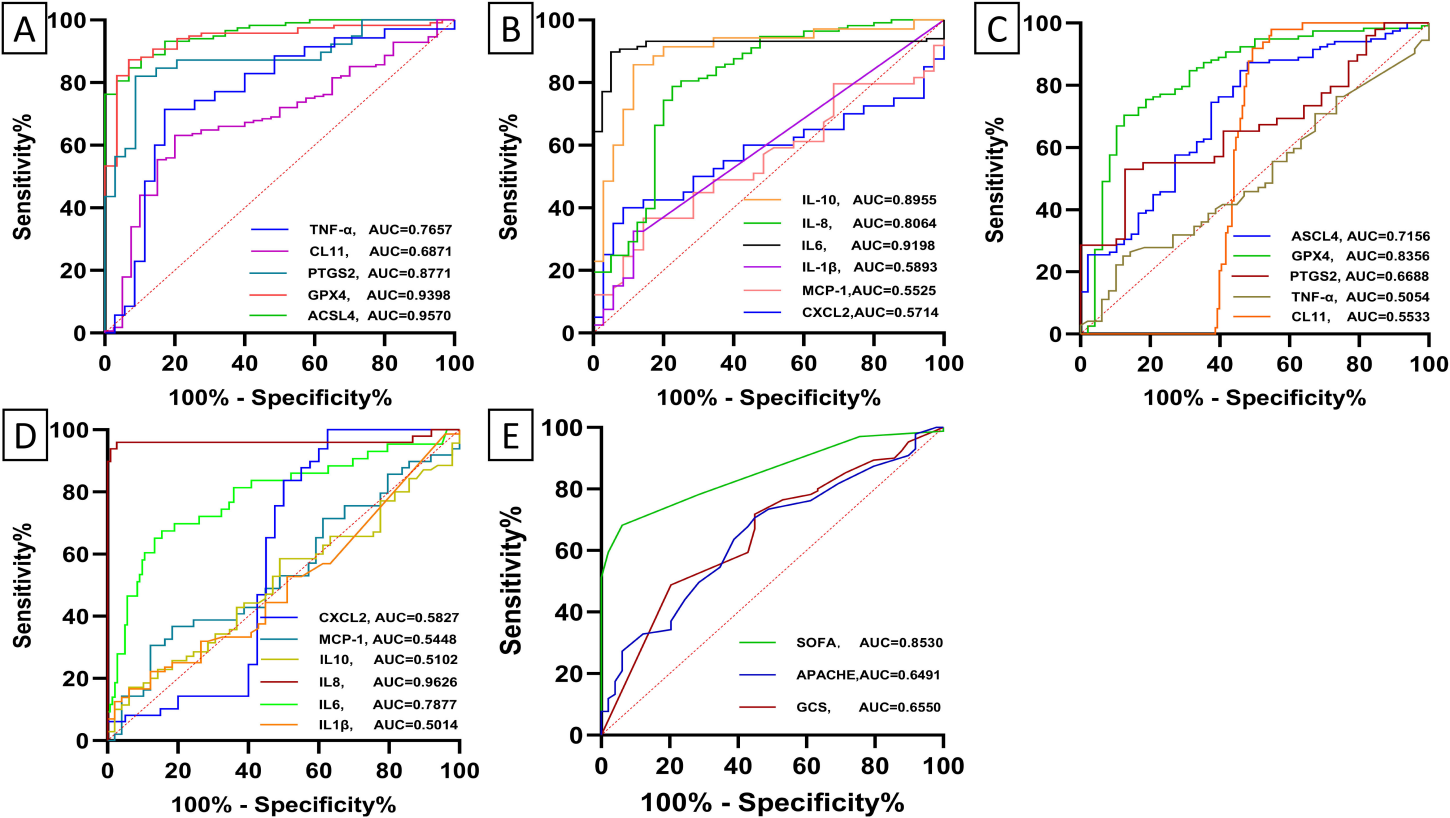
Receiver operating characteristic (ROC) curves depict the diagnostic and differential diagnostic accuracy of baseline levels of biomarker and ICU clinical scores between healthy subjects and sepsis. **(A, B)** Receiver operating characteristic (ROC) curves depict the diagnostic accuracy of baseline levels of biomarkers between healthy subjects and sepsis. ACSL4 (95%CI:0.93-0.99, p-value < 0.0001), GPX4 (95%CI:0.90-0.98, p-value < 0.0001), PTGS2(95%CI:0.79-0.96, p-value < 0.0001), CL11(95%CI:0.60-0.77, p-value = 0.0002), TNF-α (95%CI: 0.65-0.88, p-value = 0.0001), CXCL2(95%CI: 0.44 -0.71, p-value = 0.288), MCP-1(95%CI:0.43-0.68, p-value = 0.414), IL1β(95%CI:0.46-0.72, p-value = 0.184), IL6 (95%CI:0.8-0.97, p-value < 0.0001), IL8 (95% CI:0.72-0.89, p-value < 0.0001), IL10(95% CI:0.82-0.98, p-value < 0.0001). **(C, D)** Receiver operating characteristic (ROC) curves depict the diagnostic accuracy of baseline levels of biomarker between sepsis and non-sepsis. ACSL4 (95%CI :0.63-0.80, p-value < 0.0001), GPX4 (95%CI:0.76-0.91, p-value < 0.0001), PTGS2 (95%CI:0.56-0.78, p-value = 0.007), CL11(95%CI: 0.48 = 0.63, p-value = 0.256), TNF-α (95%CI: 0.40-0.61, p-value = 0.920), CXCL2(95%CI: 0.45- 0.72, p-value = 0.185), MCP-1(95%CI: 0.43-0.66, p-value = 0.445), IL1β(95%CI: 0.39-0.61, p-value = 0.979) IL6 (95%CI:0.70-0.87, p-value < 0.0001), IL-8 (95%CI :0.91-1.00, p-value < 0.0001), and IL10(95% CI: 0.41-0.61, p-value = 0.850). **(E)** Receiver operating characteristic (ROC) curves depict the diagnostic accuracy of ICU clinical scores between sepsis and non-sepsis. SOFA score (95%CI: 0.80-0.90, p-value < 0.0001), APACHE II score (95%CI: 0.56-0.73, p-value = 0.002), and GCS score (95% CI: 0.57-0.74, p-value = 0.001).

**Figure 6:**
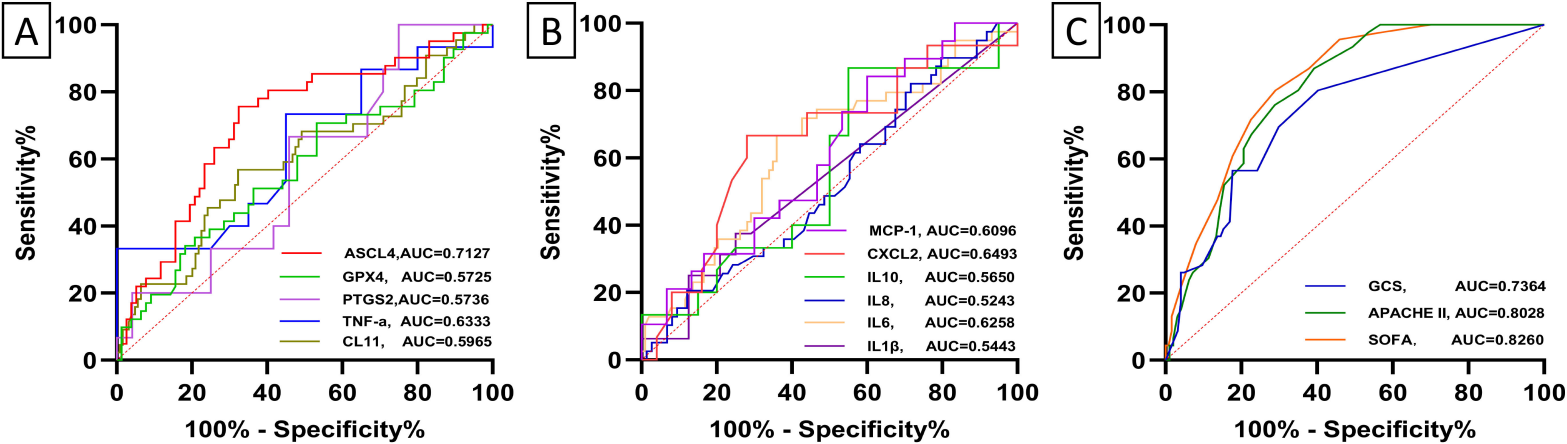
Receiver operating characteristic (ROC) curves depict the diagnostic accuracy of baseline biomarker levels and clinical scores for 28-day mortality in patients with sepsis. **(A)** ACSL4 (95% CI: 0.61-0.81, p*-*value = 0.0001), GPX4 (95%CI: 0.46-0.68, p*-*value = 0.196), PTGS2 (95%CI: 0.39-0.76, p*-*value = 0.444), CL11(95%CI: 0.49 - 0.70, p*-*value = 0.058), TNF-α (95%CI: 0.44-0.83, p*-*value = 0.182). **(B)** CXCL2(95%CI: 0.48- 0.83, p*-*value = 0.118), MCP-1(95%CI: 0.45-0.77, p*-*value = 0.200), IL1β (95%CI: 0.36-0.73, p*-*value = 0.639) IL6 (95%CI: 0.52-0.73, p*-*value = 0.021), IL-8 (95%CI: 0.41-0.64, p*-*value = 0.673), and IL10(95% CI: 0.37-0.76, p*-*value = 0.516). **(C)** SOFA score (95%CI: 0.76-0.89, p*-*value < 0.0001), APACHE II score (95%CI: 0.73-0.87, p*-*value < 0.0001), and GCS score (95% CI: 0.65-0.82, p*-*value < 0.0001).

Receiver operating characteristic (ROC) curve analysis was employed to evaluate the diagnostic and differential diagnostic potential of biochemical indicators for sepsis, septic shock, and the prediction of mortality in septic patients. The analysis revealed that several biomarkers exhibited significant diagnostic value in differentiating sepsis patients from healthy controls. Specifically, ACSL4, GPX4, IL-6, hs-CRP, PCT, PTGS2, IL-8, IL-10, WBC, and GFR demonstrated robust diagnostic performance (all p*-*value < 0.0001) (**Figures 5A, B**; **Supplementary Figure 4A**). For differentiating sepsis from non-sepsis in ICU patients, IL-8, ACSL4, GPX4, CRP, PCT, NEU%, IL-6, and PTGS2(p*-*value =0.007) exhibited significant diagnostic value (all p*-*value <0.00001) (**Figures 5C, D**; **Supplementary Figure 4B**). In predicting mortality among septic patients, ACSL4, IL-6, and NEU% were identified as the most promising biomarkers (**Figure 6**; **Supplementary Figure 5**). Across all diagnostic, differential diagnostic, and mortality prediction analyses, the SOFA score, APACHE II score, and GCS score consistently demonstrated high diagnostic values (p*-*value < 0.0001) (**Figures 5E**, **6C**).

### Correlations between ferroptosis-related proteins and standard clinical variables

Baseline levels of ACSL4 exhibited positive correlations with the SOFA score (Rho = 0.354, p*-*value < 0.0001), APACHE II score (Rho = 0.317, p*-*value < 0.0001), and the presence of septic shock (Rho = 0.274, p*-*value = 0.003), while exhibiting a negative correlation with the GCS score (Rho = -0.218, p*-*value = 0.018). Baseline GPX4 levels were positively correlated with the SOFA score (Rho = 0.204, p*-*value = 0.027) and APACHE II score (Rho = 0.233, p*-*value = 0.011). Baseline IL-6 levels were positively correlated with the SOFA score (Rho = 0.236, p*-*value = 0.005) and the presence of septic shock (Rho = 0.182, p*-*value = 0.03). Similarly, Baseline PTGS2 levels were positively correlated with the presence of septic shock (Rho = 0.317, p*-*value = 0.049).

### Ferroptosis-related proteins show potential for predicting 28-day mortality and survival outcomes in patients

**Figure 7:**
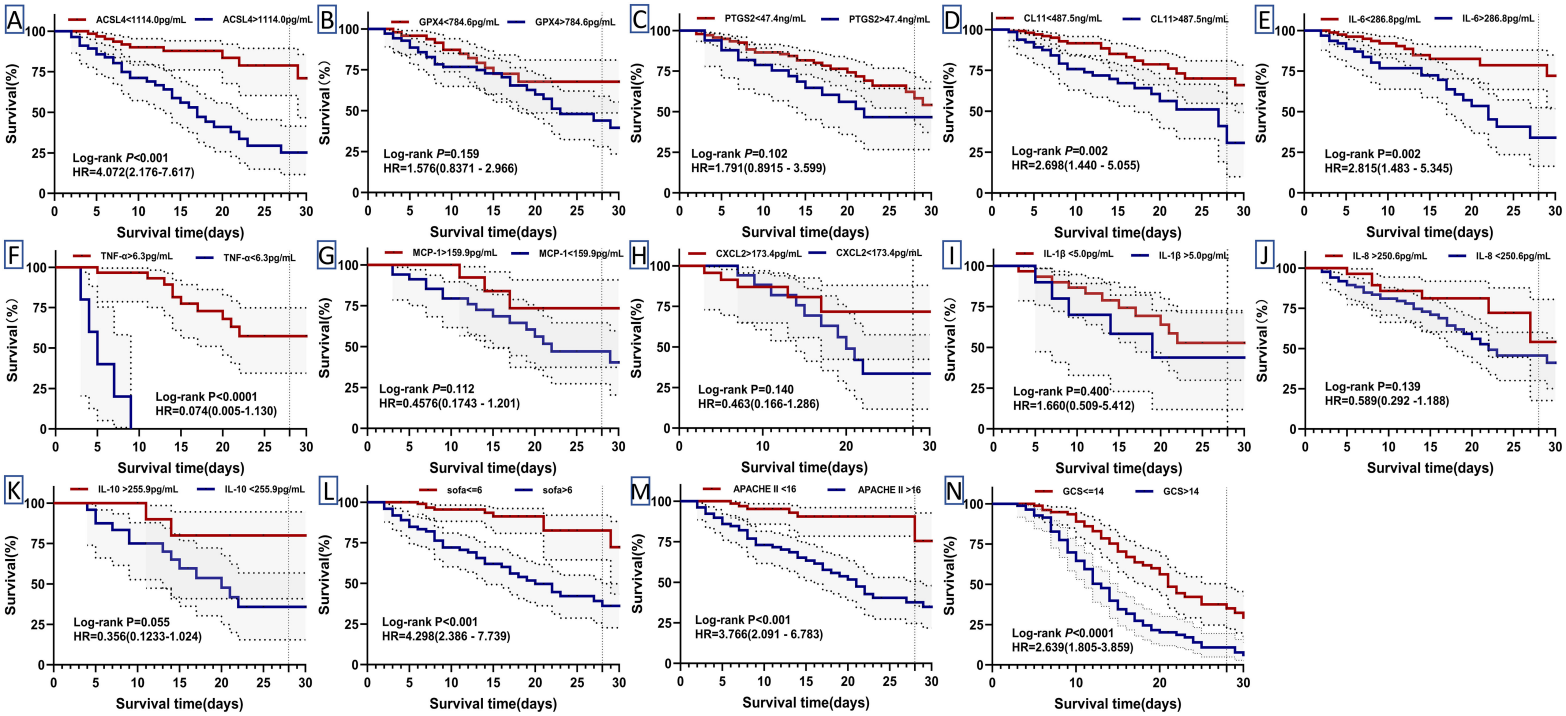
Kaplan-Meier curves for 28-day mortality in patients with sepsis. Their cut-off values divided biomarker levels and clinical scores into high and low (**Table 1**). Differences were assessed by log-rank test **(A–N)**. HR: hazard ratios. P: p-value.

Kaplan-Meier survival analysis was performed to assess the influence of independent risk factors on 28-day mortality and survival time in septic patients. The cutoff values derived from the receiver operating characteristic (ROC) curves for predicting 28-day mortality were used to dichotomize serum biomarker concentrations, SOFA score, APACHE II score, and GCS score into binary variables (**Table 1**). Death was defined as the outcome event, and the data were analyzed using the Log-Rank test. The survival curve analysis revealed that sepsis patients with elevated serum levels of ACSL4, CL-11, and IL-6 above the respective cutoff values had significantly shorter survival times (**Figures 7A, D, E**). In contrast, lower concentrations of TNF-α and neutrophil percentages (NEU%) were associated with reduced survival times (**Figures 7F**; **Supplementary Figure 6D**). Furthermore, patients with higher SOFA scores, APACHE II scores, and GCS scores exhibited shorter survival times compared to those with lower scores (**Figures 7L–N**). The serum level changes of other indicators have no significant effect on patient survival time (**Figures 7B, C, G–K**).

**Table 1:** The AUC and related indicators to predict 28-day mortality by various parameters.

Variables	AUC (95%CI)	SE (%)	SP (%)	PPV (%)	NPV (%)	Cut-off value
ACSL4(pg/mL)	0.713(0.61−0.81)	75.61	67.53	46.34	88.19	1114.0
GPX4(pg/mL)	0.573(0.46-0.68)	70.73	46.75	33.00	81.16	784.6
PTGS2(ng/mL)	0.574(0.39-0.76)	100	25.00	33.08	100.00	47.4
CL11(ng/mL)	0.599(0.49-0.70)	56.82	67.74	39.51	80.88	487.5
CXCL2(pg/mL)	0.649(0.47-0.83)	66.67	72.00	46.89	85.35	173.4
MCP-1(pg/mL)	0.610(0.45-0.77)	84.21	40.00	34.23	87.23	159.9
TNF-α(pg/mL)	0.633(0.44- 0.83)	33.33	100	100	80.18	6.3
IL1β(pg/mL)	0.544(0.36- 0.73)	31.25	79.17	35.74	75.64	5.0
IL6(pg/mL)	0.626(0.52-0.73)	66.67	64.08	40.77	83.83	286.8
IL8(pg/mL)	0.524(0.41- 0.64)	82.05	28.38	29.81	81.00	250.6
IL10(pg/mL)	0.520(0.38- 0.66)	16.67	95.24	56.49	75.5	88.9
PCT(ng/mL)	0.523(0.43-0.62)	86.05	27.64	30.60	84.24	55.4
Hs-CRP(mg/L)	0.502(0.39-0.61)	68.42	42.17	30.49	78.27	130.9
WBC(*10^9^/L)	0.511(0.40-0.62)	34.8	81.5	41.09	77.12	17.8
NEU(%)	0.658(0.57-0.75)	95.65	36.29	35.76	95.74	28.9
LA (mmol/L)	0.546(0.39- 0.70)	50.00	71.05	39.04	79.31	5.1
GFR (mL/min)	0.545(0.35-0.56)	19.6	85.5	33.39	74.15	100.4
SOFA score	0.826(0.76-0.89)	80.43	70.97	50.67	90.72	6.5
APACHE II score	0.803(0.73-0.87)	86.96	60.82	45.15	92.64	16.5
GCS score	0.736(0.65-0.82)	80.43	59.68	42.52	89.16	14.5

AUC, Area Under Curve; SE, Sensitivity; SP, specificity; PPV, positive predictive value; NPV, negative predictive value.

## Discussion

Sepsis is marked by poor treatment outcomes and high mortality rates, largely due to its unclear pathogenesis and the lack of effective early diagnostic, therapeutic, and prognostic markers. Identifying reliable biomarkers for early diagnosis and prognosis of sepsis is crucial to improving treatment efficacy and patient outcomes. Our study demonstrated that ferroptosis-related protein markers ACSL4 and GPX4 possess significant diagnostic and differential diagnostic value in sepsis, including the ability to predict 28-day mortality in our study cohort. These markers may emerge as novel serum biomarkers for diagnosis and differential diagnosis of sepsis.

Ferroptosis is a form of regulated cell death characterized by intracellular iron overload and subsequent lipid peroxidation. A substantial body of evidence highlights the significant regulatory role of ferroptosis in inflammation, sepsis, and its associated complications (18, 24, 26, 27). Growing evidence indicates that pharmacological interventions targeting ferroptosis-related molecules, such as ferroptosis inhibitors, are demonstrating therapeutic potential in sepsis (28–31). Additionally, studies have reported that ferroptosis-related genes are valuable for the early diagnosis of sepsis in pediatric populations (32, 33). Building on these findings, the present study investigates the diagnostic potential of proteins encoded by ferroptosis-related genes in adult sepsis.

GPX4 is intricately linked to ferroptosis and functions as a principal inhibitor of this process. It exerts its inhibitory role by regulating the GSS/GSR complex, highlighting its significance in modulating ferroptosis-related pathophysiological mechanisms across various diseases, including sepsis (34). ACSL4 is a pivotal lipid-metabolizing enzyme that catalyzes the esterification of long-chain polyunsaturated fatty acids (PUFAs, e.g., arachidonic acid and adrenic acid) with coenzyme A (CoA) to generate acyl-CoA derivatives. This enzymatic process is essential for regulating membrane phospholipid composition, signal transduction, and ferroptosis control (35, 36). Studies have demonstrated that ACSL4 is a key regulator of ferroptosis, with its expression level being closely correlated to cellular sensitivity to ferroptosis (37). PTGS2 is a recognized marker of ferroptosis and is widely used in both *in vitro* and *in vivo* studies. However, the upregulation of PTGS2 can also be observed under various inflammatory conditions, suggesting its potential as a biomarker for early diagnosis, treatment, and prognosis evaluation of sepsis. In this study, we detected the serum levels of ACSL4, GPX4, and PTGS2 in ICU sepsis patients, non-sepsis patients, and healthy individuals. The results confirmed that serum levels of ACSL4 and GPX4 possess high diagnostic and differential diagnostic values for sepsis, comparable to the SOFA score, CRP, and PCT, and superior to the APACHE II score and GCS score. In predicting 28-day mortality in sepsis patients, ACSL4 emerged as a better indicator than traditional infectious markers such as PCT and CRP, though it was less robust than the SOFA score and APACHE II score. A high level of ACSL4 (>1114 pg/mL) was associated with a 4-fold increase in the risk of death compared to a low level of ACSL4 (<1114 pg/mL), underscoring its prognostic significance. Notably, there were no significant differences in serum ACSL4, GPX4, and PTGS2 levels among septic patients with bacterial, fungal, and viral infections, suggesting that these levels are unaffected by the type of pathogen (**Supplementary Figure 7**). CL-11 is a pattern recognition molecule of the lectin complement pathway. It participates in the innate immune response by activating the lectin pathway of complement and is involved in the immune response and regulation of various diseases (38). Studies have shown that infection with different pathogens can induce specific expression and varying degrees of changes in CL-11 in different tissues. This study found that CL-11 levels in sepsis patients was higher than that in healthy individuals. However, ROC curve analysis revealed that CL-11 had relative low diagnostic and differential diagnostic value for sepsis. Nevertheless, a serum level of CL-11 exceeded 487.5 ng/mL demonstrated good predictive value for mortality in sepsis patients (HR = 2.698, 95% CI, 1.440-5.055, p*-*value = 0.002), consistent with previous finding (39). Discrepancies in results across studies may be attributed to the differences in the underlying characteristics of the study populations.

PTGS2 (COX-2) is primarily expressed in the parenchymal cells of various tissues and is recognized as a ferroptosis marker (20). Upon stimulation by inflammatory factors such as IL-1, TNF, LPS, cAMP, or others, the expression level of COX-2 can increase by approximately 80-fold. This upregulation promotes the production of high levels of prostaglandin-endoperoxides, thereby triggering inflammatory responses. However, few studies have investigated serum PTGS2 levels in sepsis patients. In this cohort study, serum PTGS2 levels were measured in sepsis patients, non-sepsis patients, and healthy individuals. Significant differences in PTGS2 expression were observed between sepsis patients and healthy individuals (p*-*value < 0.0001), as well as between sepsis and non-sepsis patients (p*-*value < 0.001). PTGS2 demonstrated strong diagnostic performance for sepsis (AUC = 0.8771) and was effective in differentiating sepsis patients from non-sepsis patients (AUC = 0.6688, 95% CI: 0.56–0.78, p*-*value = 0.007). However, PTGS2 did not show strong predictive value for the prognosis of sepsis. The diagnostic potential of PTGS2 highlights the important role of ferroptosis markers in the diagnosis of sepsis.

MCP-1, also known as Chemokine (CC-motif) ligand 2(CCL2), is a pivotal chemokine that promotes endothelial cell activation, and monocyte migration and regulates leukocyte function. It also mediates the release of various pro-inflammatory substances (40, 41). Elevated MCP-1 expression levels have been observed in patients with sepsis, correlating closely with organ dysfunction and mortality (42). A study by He et al. found a positive correlation between MCP-1 levels and disease severity in sepsis patients (43). However, our study found that MCP-1 did not exhibit significant value in the diagnosis, differential diagnosis, or prognosis prediction of sepsis. The reasons for this discrepancy with other studies will be further explored, which may relate to differences in baseline clinical characteristics of the study populations and the number of patients included.

Besides analyzing ferroptosis-related protein markers, this cohort study also evaluated traditional biochemical indicators of infection, including IL-1β, IL-6, IL-8, IL-10, CXCL2, and TNF-α. The results demonstrated that IL-6, PCT, CRP, IL-8, and NEU% exhibited robust diagnostic value in the diagnosis, differential diagnosis, and prognosis of sepsis. However, IL-1β, IL-10, CXCL2, and TNF-α showed limited diagnostic utility, being significant only in specific contexts. For example, IL-10 proved valuable in distinguishing sepsis patients from healthy individuals, with an AUC of 0.8955, aligning with findings from other studies (4, 44, 45). Furthermore, WBC and NEU% have emerged as highly valuable indicators for diagnosing, differentiating, and predicting the prognosis of sepsis. These results highlight the ongoing importance of traditional blood cell counts as essential diagnostic tools for infectious diseases, a diagnostic significance that should not be underestimated compared to other infectious markers (46).

To further elucidate the clinical relevance of these biomarkers, we conducted a correlation analysis between serum biomarkers and routine clinical characteristics. The baseline level of ACSL4 was positively correlated with the SOFA score (Rho = 0.354, p*-*value < 0.0001), APACHE II score (Rho = 0.317, p*-*value < 0.0001), and the presence of septic shock (Rho = 0.274, p*-*value = 0.003), but negatively correlated with the GCS score (Rho = -0.218, p*-*value = 0.018). The baseline GPX4 level was positively correlated with the SOFA score (Rho = 0.204, p*-*value = 0.027) and APACHE II score (Rho = 0.233, p*-*value = 0.011). Additionally, the baseline level of IL-6 was positively correlated with the occurrence of septic shock (Rho = 0.182, p*-*value = 0.03), as was PTGS2 (Rho = 0.317, p*-*value = 0.049). Collectively, these findings indicate that ACSL4, GPX4, and PTGS2, along with IL-6, hold significant diagnostic and prognostic value in sepsis. These biomarkers may serve as potential diagnostic markers for sepsis, thus warranting further investigation in clinical settings.

Several limitations of this study should be acknowledged. First, this was a single-center study conducted over a one-year period with a relatively small number of patients and disease types. This design may introduce potential bias from unknown confounding factors. Additionally, sepsis is a multifactorial disease with complex pathophysiological characteristics. The study population showed considerable heterogeneity in terms of pathogen types, infection sites, and comorbidities, which may have significantly influenced the immune response and thereby affected biomarker levels.

## Conclusions

In conclusion, elevated baseline serum levels of ACSL4 above the cutoff value in critical care patients were independently associated with increased 28-day mortality. Our findings suggest that ACSL4 and GPX4 may serve as more effective markers for septic shock compared to traditional inflammatory markers such as CRP and PCT in this patient population. Future larger studies that incorporate repeated measurements and focus on specific pathogens are necessary to further clarify the multifaceted pathophysiological mechanisms underlying ACSL4 and GPX4 in sepsis. Such studies will also provide a comprehensive understanding of the innate immune response in sepsis.

## Data Availability

The original contributions presented in the study are included in the article/**Supplementary Material**. Further inquiries can be directed to the corresponding author.
