# Supplementary material for: Ferroptosis-related protein biomarkers for diagnosis, differential diagnosis, and short-term mortality in patients with sepsis in the intensive care unit
Source: Front Immunol. 2025 Apr 8;16:1528986. doi: 10.3389/fimmu.2025.1528986 (PMC12011590; doi:10.3389/fimmu.2025.1528986)
Supplement: Supplementary file 1 [file DataSheet1.docx]

Supplementary Material

## 1.Supplementary Figures

**Supplementary Figure 1.** Scatter plot of serum biomarker levels and ICU clinical scores in healthy subjects (n=50), ICU patients with sepsis (n=170), and ICU patients without sepsis (n=59). Differences between median levels of (A) PCT, (B) Hs-CRP, (C) WBC, (D) NEU%, (E) GFR in the three groups were analyzed by the Student's t-test or the Mann -Whitney U test.

Footnote: ns: No statistical significance. *p-value<0.05, **p-value<0.001, ***p-value<0.0001, ****p-value<0.00001

**Supplementary Figure 2.** Receiver operating characteristic (ROC) curves depict the diagnostic and differential diagnostic accuracy of baseline levels of biomarkers between healthy subjects and sepsis. (A) Receiver operating characteristic (ROC) curves depict the diagnostic accuracy of baseline levels of biomarkers between healthy subjects and sepsis. Hs-CRP (95%CI: 0.98-1.00, P<0.0001), PCT (95%CI: 0.97-1.00, p-value<0.0001), WBC (95%CI: 0.83-0.92, p-value<0.0001), NEU% (95%CI: 0.68-0.81, p-value<0.0001), GFR (95%CI: 0.85-0.94, p-value<0.0001). (B) Receiver operating characteristic (ROC) curves depict the diagnostic accuracy of baseline levels of biomarker between sepsis and non-sepsis. Hs-CRP (95%CI: 0.70-0.86-, p-value<0.0001), PCT (95%CI: 0.78-0.91, p-value<0.0001), WBC (95%CI: 0.47-0.64, p-value=0.266), NEU% (95%CI: 0.81-0.90, p-value<0.0001), GFR (95%CI: 0.53-0.71, p-value=0.015).

**Supplementary Figure 3**. Box plot of serum biomarker levels and ICU clinical scores in patients with septic shock (n = 69) and septic non-shock (n = 101). Differences between median levels of(A) LA, (B) Hs-CRP, (C) PCT, (D)WBC, (E) NEU% and (F) GFR in the two groups were analyzed by the Student's t-test or the Mann -Whitney U test.

Footnote: ns： No statistical significance. *p-value<0.05, **p-value<0.001, ***p-value<0.0001, ****p-value<0.00001

**Supplementary Figure 4**. Scatter plots of serum biomarker levels and ICU clinical scores in sepsis survivors (n = 124) and sepsis non-survivors (n = 46). Differences between median levels of (A) PCT, (B) Hs-CRP, (C) WBC, (D) NEU%, (E) LA and (F) GFR in the two groups were analyzed by the Student's t-test or the Mann -Whitney U test.

Footnote: ns： No statistical significance. *p-value<0.05, **p-value<0.001, ***p-value<0.0001, ****p-value<0.00001

**Supplementary Figure 5.** Receiver operating characteristic (ROC) curves depict the diagnostic accuracy of baseline biomarker levels for 28-day mortality in patients with sepsis. Hs-CRP (95%CI: 0.39-0.61, p-value=0.978), PCT (95%CI: 0.43-0.62, p-value=0.658), WBC (95%CI: 0.40-0.62, p-value=0.832), NEU% (95%CI: 0.57-0.75, p-value=0.002), GFR (95%CI: 0.44-0.65, p-value=0.369) and LA (95% CI: 0.39-0.70, p-value=0.549).

**Supplementary Figure 6**. Kaplan-Meier curves for 28-day mortality in patients with sepsis. Their cut-off values divided biomarker levels and clinical scores into high and low (Table 1). Differences were assessed by log-rank test. (A)PCT, (B)Hs-CRP, (C)WBC, (D)NEU%, (E)LA, (F)GFR.

**Supplementary Figure 7.** Expression levels of serum biomarkers under different pathogenic infections.

Footnote: ns: No statistical significance. *p-value<0.05, **p-value<0.001, ***p-value<0.0001, ****p-value<0.00001

1. Supplementary Tables

**Supplementary Table 1**. Factors associated with 28-day mortality by univariate analysis in adult patients with sepsis

| Variables | Non-survivor（n=46） | survivor（n=124） | p-value |
| --- | --- | --- | --- |
| **Basic Characteristics** |  |  |  |
| Age（years） | 66（58-74） | 58（52-70） | 0.062 |
| Dender(female，%) | 17(36.96) | 59(47.58) | 0.130 |
| **Length of hospital stay（days）** | **9（6-17）** | **14（10-22）** | **0.007** |
| Smoking **(n, %)** | 18(39.13) | 39(31.45) | 0.252 |
| Drinking **(n, %)** | 13(28.26) | 39(31.45) | 0.688 |
| Systolic blood pressure | 118（102-135） | 115（98-133） | 0.590 |
| Diastolic blood pressure | 69（62-80） | 72（62-82） | 0.629 |
| Admission temperature | 36.7（36.5-37.0） | 36.7（36.5-37.4） | 0.145 |
| **Microorganism (n, %)** |  |  |  |
| Bacterial infections | 25(54.35) | 80(64.52) | 0.078 |
| Fungal infections | 15(32.61) | 21(16.94) | 0.101 |
| **Viral infection** | **6(13.04)** | **7(5.65)** | **0.008** |
| COVD-19 | 5(10.87) | 3(2.42) | 0.063 |
| **ICU scoring systems*** |  |  |  |
| **SOFA score** | **9（7-11）** | **4（2-7）** | **<0.001** |
| **APACHE II score** | **23（18-27）** | **15（10-19）** | **<0.001** |
| **GCS score** | **11（5-14）** | **15（13-15）** | **<0.001** |
| **Underlying diseases (n, %)** |  |  |  |
| **MODS** | **16(34.78)** | **20(16.13)** | **0.011** |
| **ARDS** | **23(50.00)** | **11(8.87)** | **<0.001** |
| Chronic/acute renal failure^a^ | 19(41.30) | 47(37.90) | 0.882 |
| **Diabetes** | **11(23.91)** | **47(37.90)** | **0.046** |
| Hypertension | 22(47.83) | 49(39.52) | 0.378 |
| **Tumor^#^** | **2(4.35)** | **1(0.81)** | **0.041** |
| Cardiovascular disease | 3(6.52) | 9(7.26) | 0.762 |
| **Respiratory dysfunctiona^b^** | **26(56.52)** | **36(29.03)** | **0.002** |
| Gastrointestinal diseases^c^ | 14(30.43) | 45(36.29) | 0.700 |
| Neurological diseases | 2(4.35) | 11(8.87) | 0.287 |
| Severe trauma | 3(6.52) | 8(6.45) | 0.854 |
| Immune diseases | 1(2.17) | 3(2.42) | 0.598 |
| Urinary tract infection | 5(10.87) | 33(26.61) | 0.082 |
| **Hematologic(nonmalignant)^#^** | **2(4.35)** | **1(0.81)** | **0.041** |
| **Risk factors (n, %)** |  |  |  |
| **Septic shock** | **29(63.04)** | **40(32.26)** | **<0.001** |
| Invasive procedures | 40(86.96) | 94(75.81) | 0.158 |
| Broad-spectrum antibiotics | 34(73.91) | 84(67.74) | 0.249 |
| **Mechanical ventilation** | **33(71.74)** | **20(16.13)** | **<0.001** |
| **Vasopressor drugs** | **27(58.70)** | **29(23.39)** | **<0.001** |

*Clinical scores are not risk factors and are only used for statistical analysis and not included in multivariable logistic regression analysis.

^#^Because the number was very small, the result of multivariable logistic regression analysis is unreliable.

^a^ Chronic/Acute renal failure is the permanent or sudden and often temporary loss of kidney function with N waste retention and hypourocrinia.

^b^Includes the following diseases: chronic obstructive pulmonary disease and acute respiratory distress syndrome.

^c^ Includes the following diseases: cholecystitis, pancreatitis and peritonitis.
