# Supplementary figures and images for: Ferroptosis-related protein biomarkers for diagnosis, differential diagnosis, and short-term mortality in patients with sepsis in the intensive care unit
Source: Front Immunol. 2025 Apr 8;16:1528986. doi: 10.3389/fimmu.2025.1528986 (PMC12011590; doi:10.3389/fimmu.2025.1528986)

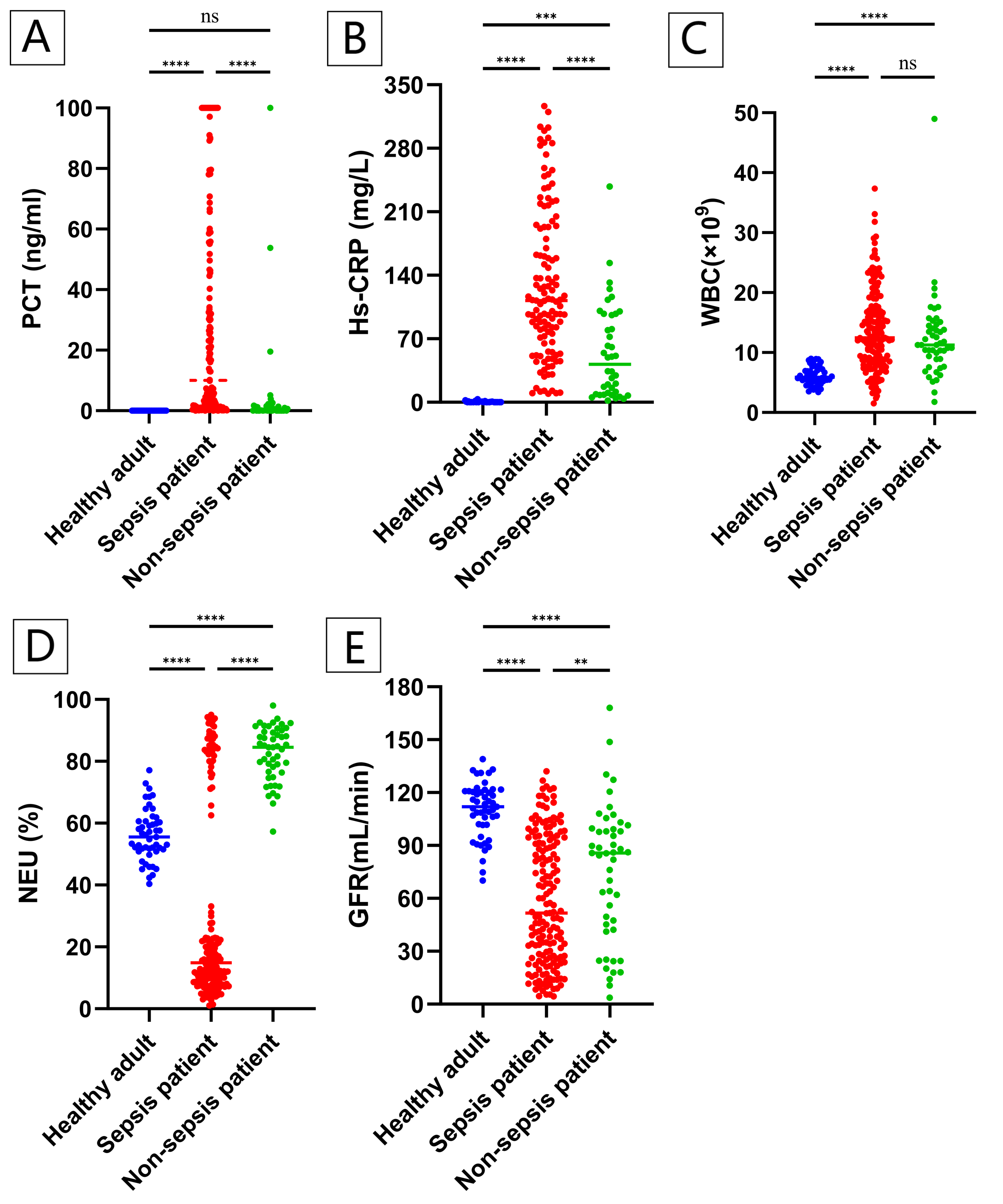

Supplement: Supplementary file 2 [file Image1.tif]

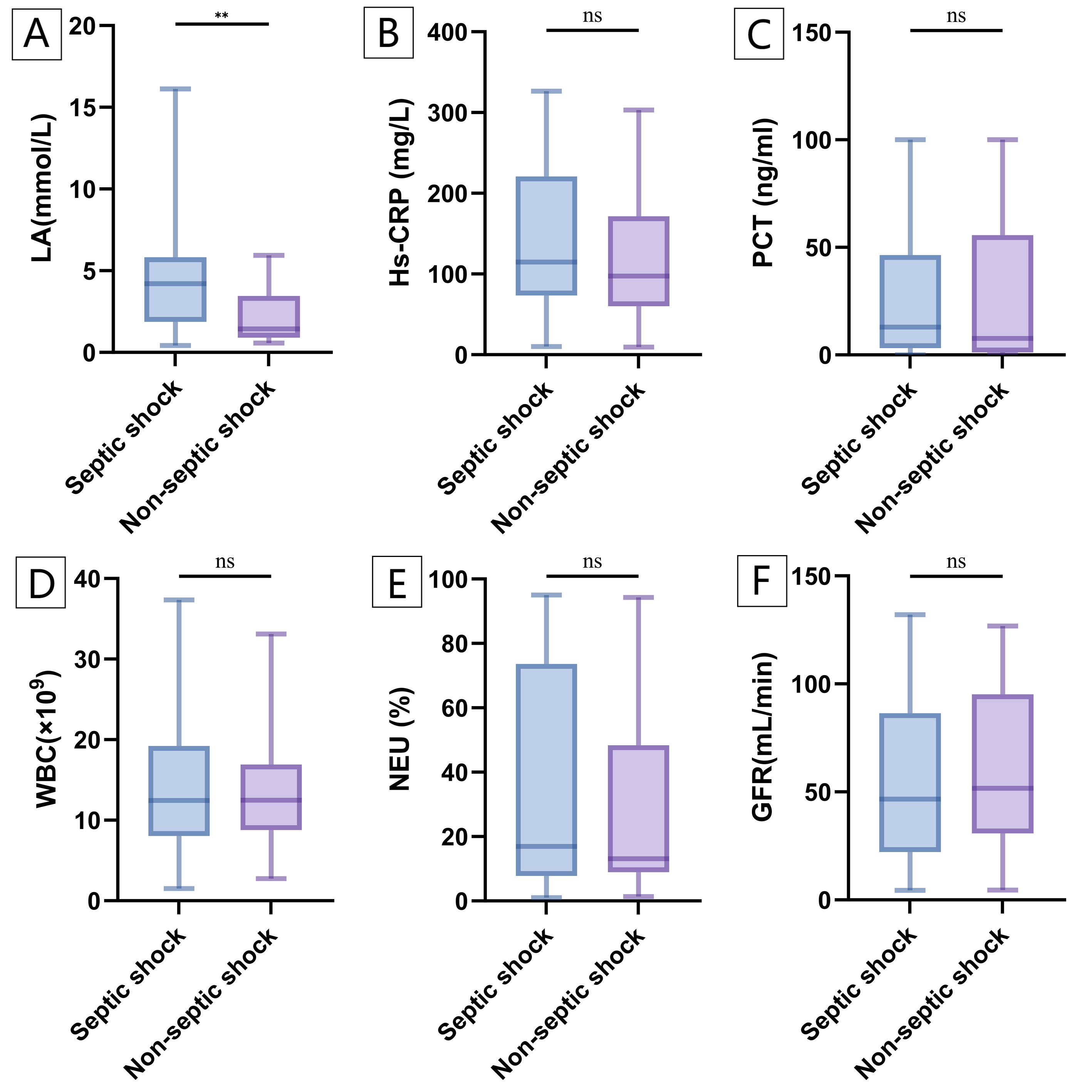

Supplement: Supplementary file 3 [file Image2.tif]

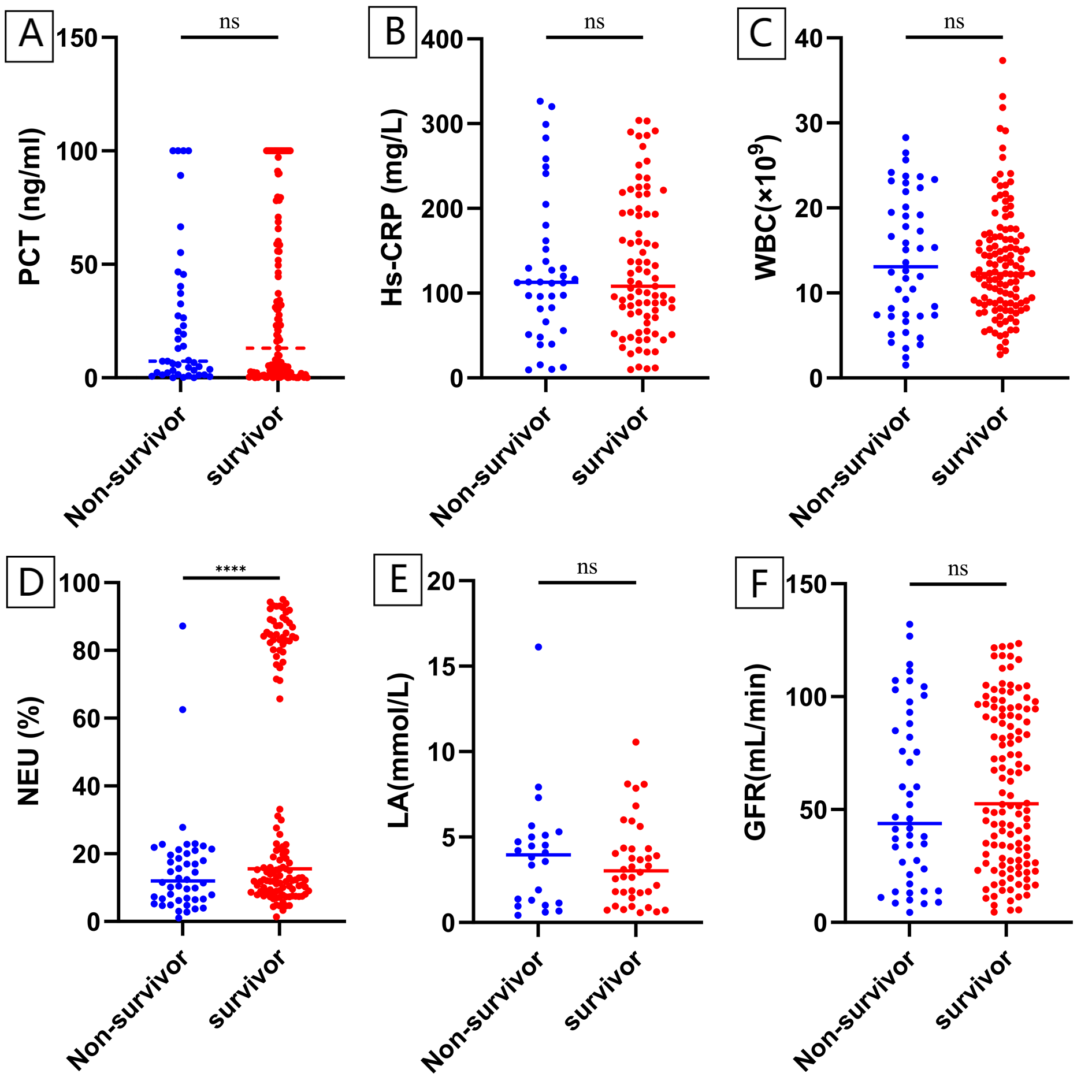

Supplement: Supplementary file 4 [file Image3.tif]

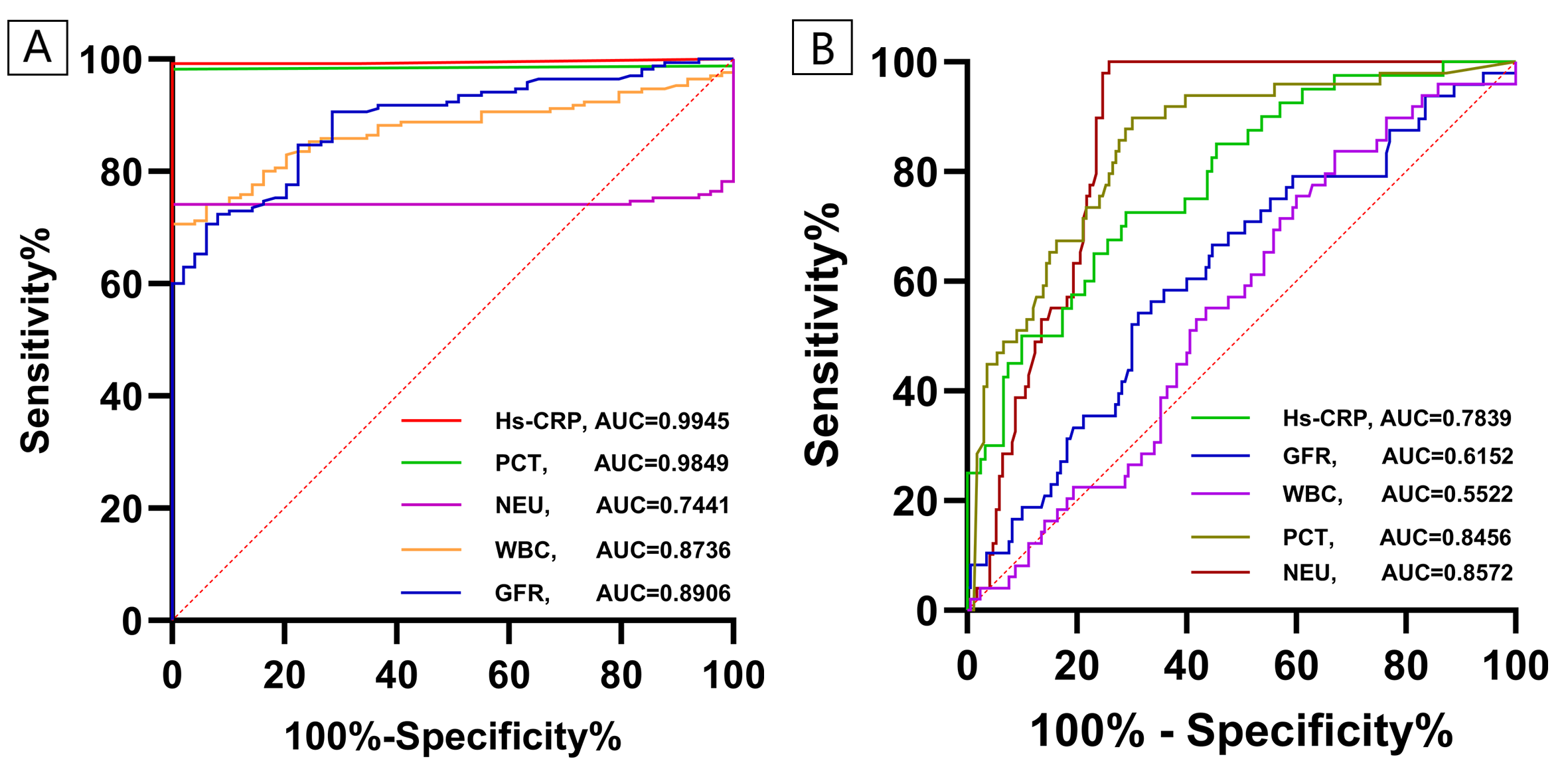

Supplement: Supplementary file 5 [file Image4.tif]

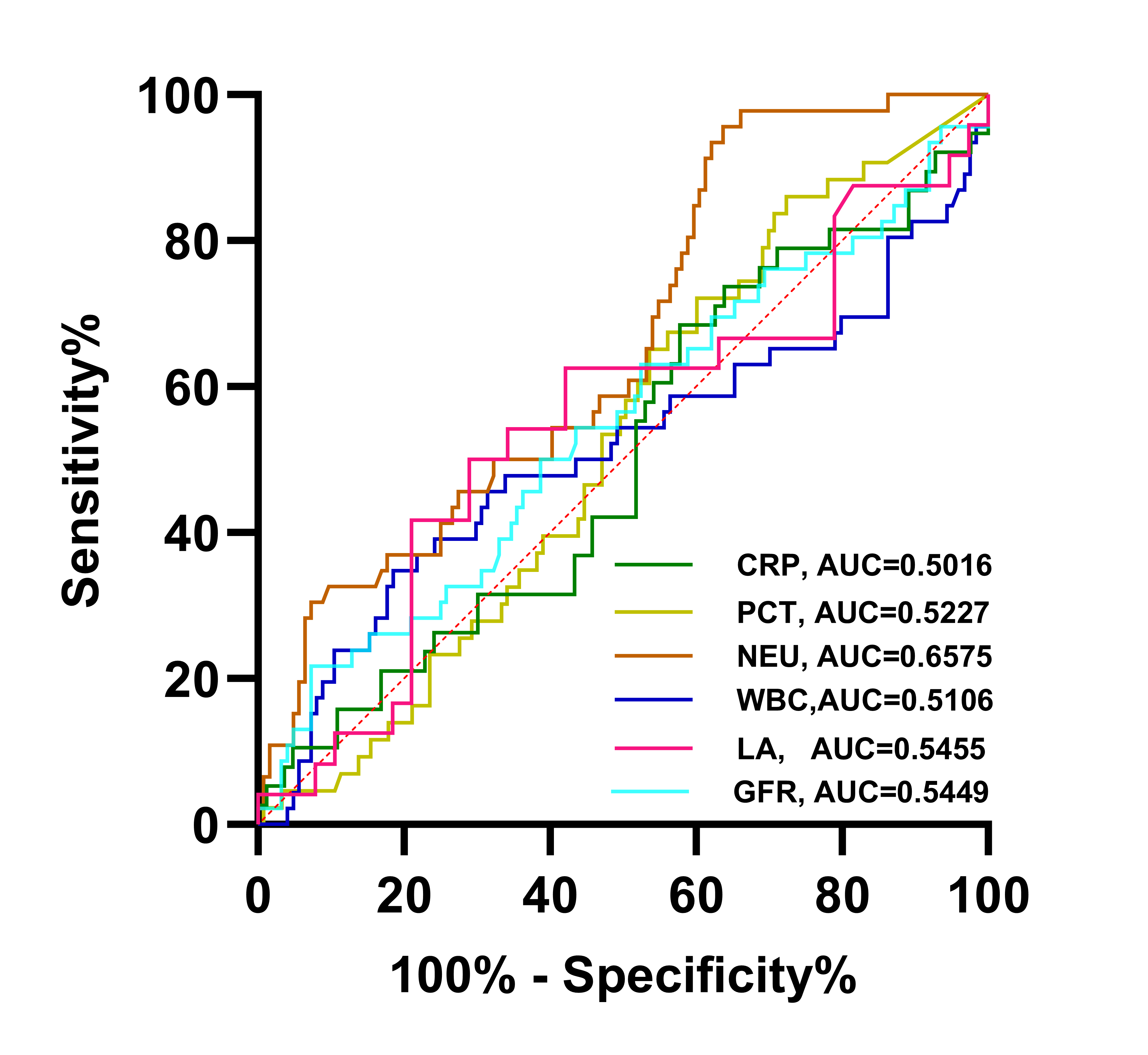

Supplement: Supplementary file 6 [file Image5.tif]

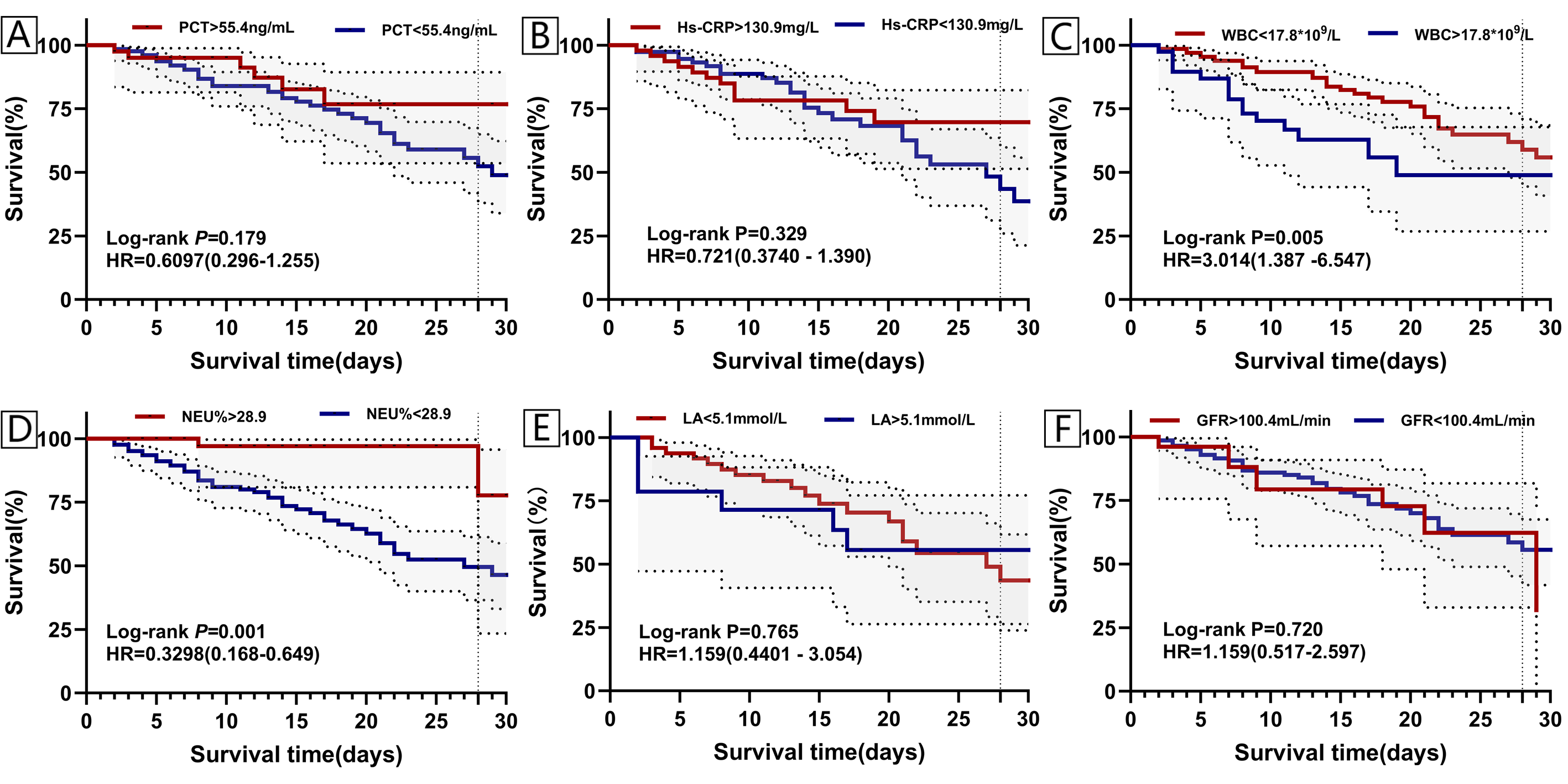

Supplement: Supplementary file 7 [file Image6.tif]

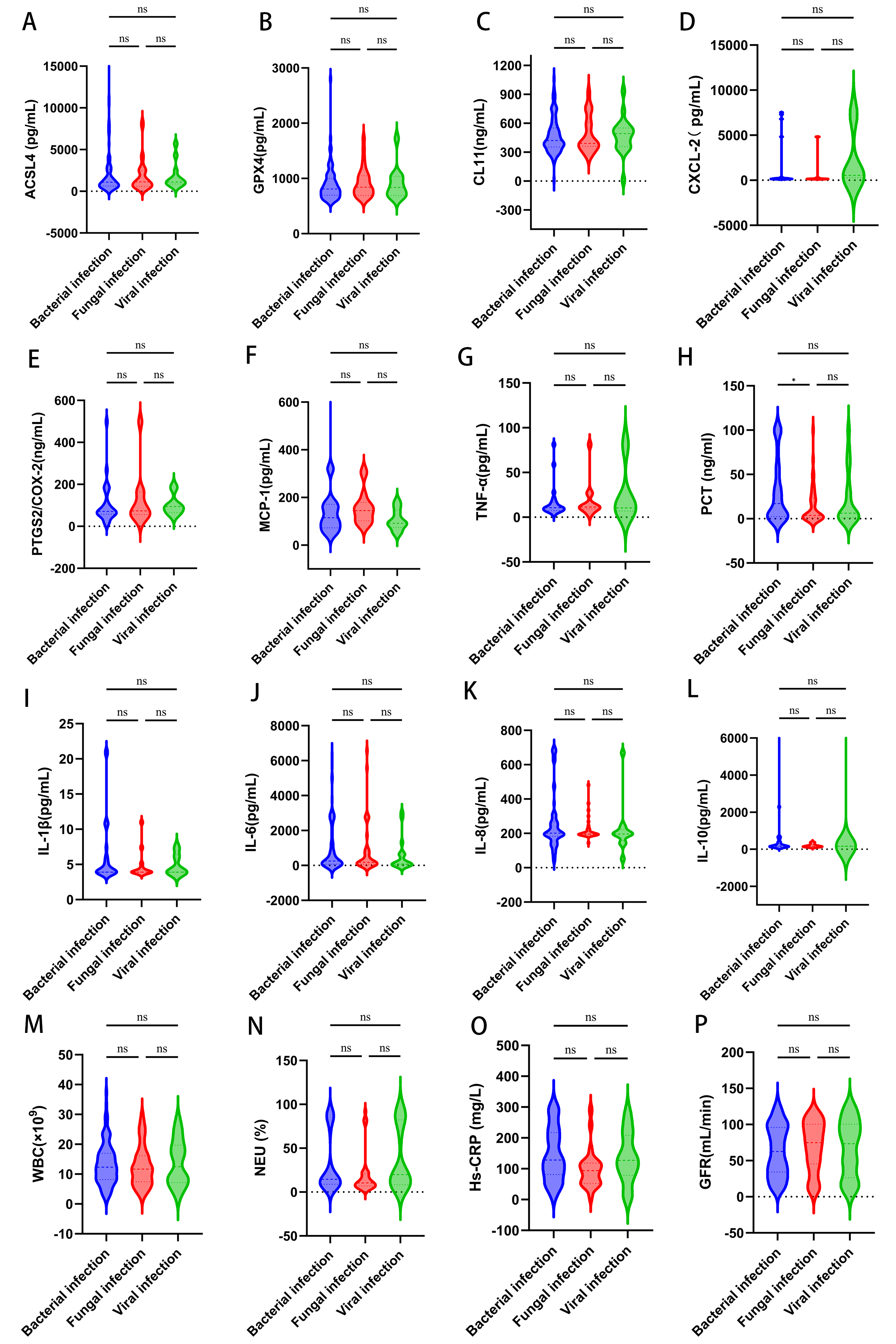

Supplement: Supplementary file 8 [file Image7.tif]
